# Oosorption in response to poor food: complexity in the trade-off between reproduction and survival

**DOI:** 10.1002/ece3.4

**Published:** 2011-09

**Authors:** Patricia J Moore, Alfredo Attisano

**Affiliations:** Centre for Ecology and Conservation, College of Life and Environmental Science, University of ExeterPenryn, Cornwall, UK

**Keywords:** Energy allocation, nutrition, oosorption, ovarian apoptosis, reproductive investment

## Abstract

Plasticity in reproductive physiology is one avenue by which environmental signals, such as poor quality food, can be coordinated with adaptive responses. Insects have the ability to resorb oocytes that are not oviposited. Oosorption is proposed to be an adaptive mechanism to optimize fitness in hostile environments, recouping resources that might otherwise be lost, and reinvesting them into future reproductive potential. We tested the hypothesis that oosorption is an evolved mechanism by which females can reallocate resources from current reproductive effort to survival and future reproduction, when conditions for reproduction are poor, by examining the reproductive physiology and life-history outcome under poor quality food in populations of the milkweed bug (*Oncopeltus fasciatus*) that have adapted to live on sunflower seed. Females fed a diet of pumpkin seeds, known to be a poor host food, had higher levels of ovarian apoptosis (oosorption), lower reproductive output, but no reduction in life span under poor nutrition, as predicted under the oosorption hypothesis. However, the schedule of reproduction was surprising given the “wait to reproduce” assumption of oosorption as early fecundity was unaffected.

## Introduction

One of the fundamental challenges for research in biodiversity is to understand how organisms respond to changing or novel environments. In particular, how do organisms meet the challenge of suboptimal, stressful, or atypical environments? We expect animals to have evolved mechanisms to balance the conflicting energy requirements for reproduction and survival (Bell and Koufopanou 1986; Stearns 1989; Messina and Fry 2003; Partridge et al. 2005; Cox et al. 2010; Stoltz et al. 2010). Under suboptimal environmental conditions, such as reduced or poor quality food, it is assumed that any energy saved by a reduction in reproduction can be used to increase survival, deferring reproduction in order to survive until conditions improve.

Insects, like many animals, have the ability to respond plastically to environmental stress, resorbing oocytes that are not oviposited (Bell and Bohm 1975), an adaptive mechanism to optimize fitness in hostile environments by recouping resources that might otherwise be lost (Bell and Bohm 1975; Papaj 2000; Barrett et al. 2008; Boggs 2009). These resources can then be reinvested into somatic functions that increase life span and future reproductive potential. Thus, the ability to resorb eggs provides the opportunity to plastically respond to varied environmental conditions throughout a reproductive lifetime.

The predicted positive phenotypic correlation between egg resorption and longevity has been documented, but is somewhat weak. Most studies of oosorption in insects focus on host plant availability and quality (Awmack and Leather 2002). For example, in butterflies, a reduction in food leads to a reduction in fecundity, accompanied by an increase in oocyte resorption, but no reduction in life span (Boggs and Ross 1993). In ladybird beetles, a reduction in host plant availability, resulting in low oviposition due to egg resorption, is correlated to periods of increased survival in females but not in males (Ohgushi 1996). The conclusions drawn from both of these studies depend on the correlation between egg resorption and subsequent female survival. They suggest, but do not demonstrate, a trade-off between reproduction and life span mediated by the recycling of nutrients invested in oocytes that cannot be used.

The oosorption hypothesis is underpinned by the “Y model” (van Noordwijk and de Jong 1986; King et al. 2011), in which negative correlations among traits such as reproduction and longevity and current versus future reproduction arise through competition for limiting resources. Under food or host stress, oosorption will lead to nutrients being redirected from eggs to somatic maintenance. While there is support for the Y mode’(e.g. King et al. 2011), recent work on the molecular mechanisms underlying the trade-off between reproduction and survival demonstrates that it cannot be fully understood through simple competition for resources between reproduction and somatic maintenance (Partridge et al. 2005; Boggs 2009; Flatt and Schmidt 2009; Stearns 2011). Studies are beginning to address the physiological nature of these trade-offs in natural populations (Cox et al. 2010; Stotz et al. 2010). Their results emphasize the potential complexity underlying the trade-off within and across taxa and illustrate the need to examine these in multiple species and environments.

We investigated the potential for oosorption to play a role in the response to novel food in the milkweed bug,*Oncopeltus fasciatus*. The evolutionary ecology of North American populations of*O. fasciatus*has been well documented. Northern populations of*O. fasciatus*are migratory; they overwinter in southern states and migrate north in spring with the flowering and seed set of the host plant*Asclepias syriaca*(common milkweed; Palmer and Dingle 1986; Dingle et al. 1988; Leslie 1990; Dingle 1992) and are adapted to an abundant and reliable food supply (Palmer and Dingle 1986). Florida populations show a greater variance in migratory and diapause behavior (Dingle et al. 1980) and show an increased level of host acceptance, having the ability to feed on alternative hosts when*Asclepias*seeds are temporarily not available (Klausner et al. 1980). Puerto Rican populations, on the other hand, are nonmigratory and adapted to an ephemeral and limited food supply as their host plant,*A. curassavica*, provides fewer seeds and is cleared by farmers (Dingle et al. 1980). These adaptations are evident in comparisons to the genetic architecture between populations. Iowa bugs show a “migratory syndrome” with genetic correlations among body size, wing length, flight capacity, and early fecundity (Palmer and Dingle 1986). These genetic correlations are not present in the Puerto Rico populations (Dingle et al. 1988).

Although*O. fasciatus*preferentially uses milkweed when available, a general ability to adapt to new host plants for food has also been shown using experimental evolution.*Oncopeltus fasciatus*can be reared on a variety of food sources including sunflower, cashew and pumpkin seeds, and peanuts (Beck et al. 1958; Gordon and Gordon 1971; Feir 1974). Although initial performance on these alternative hosts is reduced in comparison to performance on milkweed, experimental evolution imposed by exclusive use of these hosts for more than 10 generations leads to improved performance (Gordon and Gordon 1971; Feir 1974).

To study the role of oosorption in responding to a poor food source use, we used a laboratory strain derived from the Iowa population that has undergone experimental evolution and is adapted to live on sunflower seeds. The population we used for this study has been in culture for over 45 years and reared exclusively on sunflower seeds, which corresponds to over 400 generations of artificial selection. This population appears to have expanded its host range, as it performs well on either sunflower or milkweed. Using a novel food, pumpkin seed, to manipulate food quality, we tested the prediction that reduced food quality leads to increased oosorption, decreased reproductive output, and no reduction in life span. Because investment in reproduction can include both the production of gametes and mating effort (Stoltz et al. 2010), we measured the impact of diet on the physiological mechanism of oosorption (ovarian apoptosis), reproductive output and life span of females, as well as the rate of sexual maturation and mating behavior. As a control, we also asked whether females that have adapted to a new food source have retained their ability to utilize the ancestral food, milkweed seeds.

## Materials and Methods

### Animal husbandry and experimental set-up

**Figure 1:**
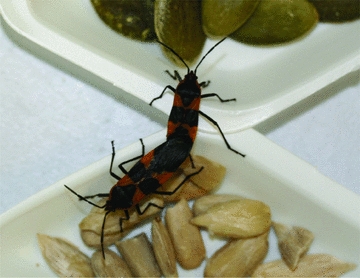
A mating pair of milkweed bugs (*Oncopeltus fasciatus*).

We obtained sunflower-adapted*O. fasciatus*cultures (Fig. 1) from Carolina Biological Supply (Burlington, NC). We housed colonies at 24°C on a 16/8-hour light/dark cycle. Cultures were supplied with ad libitum sunflower seeds and deionized water, and absorbent cotton wool as an oviposition site. We selected newly emerged adults daily from a culture of late instar nymphs. On the day of adult emergence, we placed one female and one male in a Petri dish supplied with a cotton dental wick wetted with deionized water, absorbent cotton wool for an oviposition site, and ad libitum food. Half of the pairs received organic, unsalted sunflower seeds (adapted diet) and the other half received organic, unsalted pumpkin seeds (novel diet; Goodnessdirect.co.uk, Daventry, Northamptonshire, UK) as a food source. Forty-four pairs were set up on pumpkin seed and 41 pairs were set up on sunflower seed.

In a separate experiment, we repeated this design using the same stock population but divided the pairs between organic, unsalted sunflower seeds and their ancestral food, milkweed seeds (Educational Science, League City, TX). We followed the same rearing protocols and experimental design as described above but our samples sizes were 24 milkweed seed pairs and 27 sunflower seed pairs.

### Effect of diet on development of sexual maturation, mating rate, fecundity, and life span

We checked Petri dishes daily. The first date that copulation was observed in a dish was recorded as a measure of sexual maturation. For the pumpkin versus sunflower experiments, we also recorded daily copulation rates in pairs over 5 consecutive days, from 14 to 19 days postadult emergence. We chose this time period to ensure that all pairs had reached sexual maturity and were not yet senescing. Every day at the same time, we recorded which pairs were observed in copula. For the milkweed versus sunflower experiment, we observed pairs daily until first observed mating, and then at regular intervals over the females’ life spans. We also recorded the dates that eggs were first observed in the dish (typically on the cotton wool oviposition site, but occasionally eggs were found in the food dish), and the date that newly hatched offspring were first observed.

We provided pairs with food and water as needed and recorded the date of death of the female in the pair. Occasionally a male died and then that male was replaced with random male of a similar age that had been fed the treatment diet of his partner. Once per week following the hatching of the first offspring, we replaced the dental wick, food dish, and cotton wool oviposition site. We determined reproductive success for each pair by counting the number of eggs present on the cotton wool. Because the first cotton wool was not removed until after the first offspring hatched, data for the first reproductive event included both the number of hatchlings and the number of eggs. Under the culture conditions used, eggs took approximately one week to hatch so the subsequent oviposition sites mainly contained eggs at various stages of development although newly hatched offspring were present on occasion. Thus, reproduction can be examined over the life span of the female, and data include the weekly production of eggs from first reproduction until death.

### Ovarian apoptosis

A subset of the pairs housed on pumpkin and sunflower was used to examine female ovarian apoptosis at 10 days postadult emergence. We dissected ovaries from females and stained them using the Vybrant Apoptosis Assay kit #4 (Molecular Probes, Invitrogen, Eugene, OR) as described by Moore and Sharma (2005). This stain contains two dyes: the dye YO-PRO-1 (green fluorescence) can enter apoptotic cells but is excluded from healthy cells, while propidium iodide (red fluorescence) cannot enter living or apoptotic cells due to its large molecular size and thus only stains cells that are either necrotic or in the late stages of apoptosis (Willingham 1999; Moore and Sharma 2005). Healthy cells are unstained; apoptotic oocytes are green; and oocytes in the late stages of apoptosis or that are necrotic are red (Moore and Sharma 2005). We examined our slides using an Olympus BX51 epifluorescence microscope (Olympus UK Ltd., London, UK). For each female, we examined 10 ovarioles and counted the number of ovarioles that displayed either green or red fluorescence. We did not observe any ovarioles that showed exclusively red stain, indicating necrosis or tissue damage due to dissection. Therefore the data collected were the number of ovarioles of the 10 observed that showed evidence of apoptosis (green and red fluorescence). Staining was done blind relative to food treatment by coding females just prior to dissection. Codes were only revealed after scoring the staining levels of the ovaries.

### Data analysis

We used JMP version 5.0.1a (SAS Institute, Marlow, Buckinghamshire, UK) for our statistical analyses. Except where specified, data were analyzed using analysis of variance (ANOVA). We used repeated measures ANOVA to analyze the change in reproductive output over time between the pumpkin and sunflower seed treatments. The repeated measures output provide information on single degree of freedom contrasts, it does not provide focused pairwise comparisons. As we had a priori specific comparisons we wished to make, contrast analysis of paired comparisons is appropriate. Therefore, we tested specific hypotheses using paired*t-*tests to examine our a priori pairwise contrasts between subsequent ages (Rosenthal and Rosnow 1985). The difference in female longevity between the two treatments was examined using a Wilcoxon rank sum test.

## Results

### Effect of novel versus adapted diet on development of sexual maturation, mating rate, and egg quality

**Figure 2:**
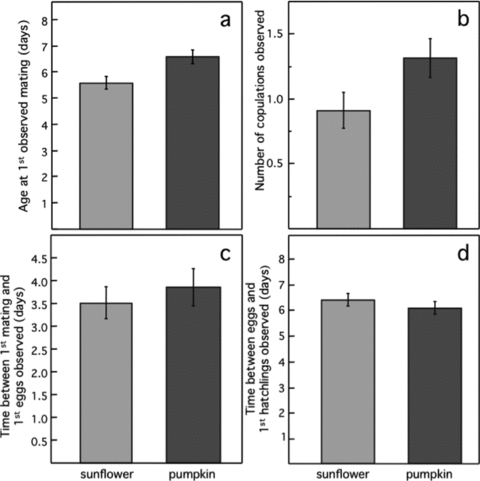
The effect of novel versus adapted diet on development of sexual maturation, mating rate, and egg quality. The effect of pumpkin seed (dark gray bars) or sunflower seed (light gray bars) is compared for mean number of days it took for pairs to become sexually mature (A), mean number of copulations observed over 5 days (B), mean number of days it took females to produce a first clutch of eggs following mating (C), and mean development time of offspring (D). Error bars represent ± standard error.

Pairs of individuals fed the novel diet of pumpkin seed took, on average, about one day longer to develop sexual maturity, as measured as the mean number of days postadult emergence when first copulations were observed (Fig. 2A,*F*_1,78_ = 7.696,*P* = 0.007). Once sexually receptive, however, pumpkin-fed pairs are more likely to be observed in copula than sunflower-fed pairs (Fig. 2B;*F*_1,89_ = 4.17,*P* = 0.044) when observed between 14 and 19 days postadult emergence.

Once females had mated, they developed and laid the initial group of eggs at the same rate in both pumpkin- and sunflower-fed pairs, measured as the mean number of days between first mating and first eggs observed (Fig. 2C;*F*_1,78_ = 0.402,*P* = 0.528). There also was no difference in development rate between the eggs laid by pumpkin-fed females and sunflower-fed females, measured as the mean number of days between appearance of the first eggs and appearance of the first hatchlings (Fig. 2D;*F*_1,73_ = 0.935,*P* = 0.337). Combined, this resulted in pumpkin-fed pairs producing their first hatchlings with a delay of about one day, with newly hatched offspring observed in pumpkin-fed pairs at a mean of 16.44 days postadult emergence, and in sunflower-fed pairs at a mean of 15.55 days postadult emergence (*F*_1,75_ = 10.147,*P* = 0.002).

### Effect of ancestral versus adapted diet on development of sexual maturation, mating rate, and egg quality

**Figure 3:**
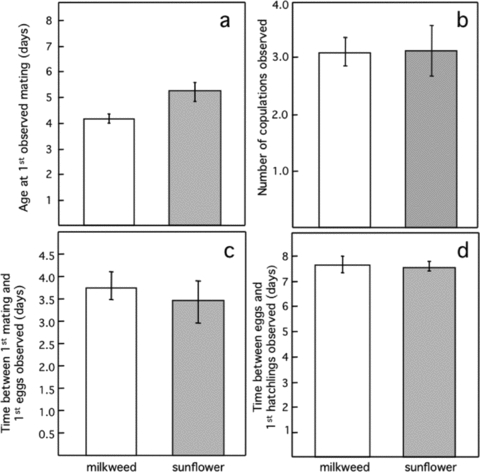
The effect of ancestral versus adapted diet on development of sexual maturation, mating rate, and egg quality. The effect of milkweed seed (open bars) or sunflower seed (light gray bars) is compared for mean number of days it took for pairs to become sexually mature (A), mean number of copulations observed (B), mean number of days it took females to produce a first clutch of eggs following mating (C), and mean development time of offspring (D). Error bars represent ± standard error.

Pairs of individuals fed the ancestral diet of milkweed seed took, on average, about one day less to develop sexual maturity, as measured by the mean number of days postadult emergence when first copulations were observed (Fig. 3A;*F*_1,54_ = 5.809,*P* = 0.019). Once sexually receptive, however, milkweed- and sunflower-fed pairs are equally likely to be observed in copula (Fig. 3B;*F*_1,54_ = 0.001,*P* = 0.973) when observed at regular intervals over their life span.

Once females have mated, they developed and laid the initial group of eggs at the same rate in both milkweed- and sunflower-fed pairs, measured as the mean number of days between first mating and first eggs observed (Fig. 3C;*F*_1,46_ = 0.480,*P* = 0.492). There also was no difference in development rate between the eggs laid by milkweed-fed females and sunflower-fed females, measured as the mean number of days between appearance of the first eggs and appearance of the first hatchlings (Fig. 3D;*F*_1,48_ = 0.111,*P* = 0.740).

### Effect of novel versus adapted diet on levels of oosorption

**Figure 4:**
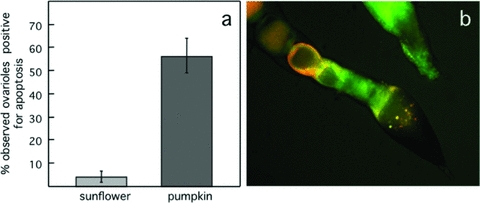
The effect of novel versus adapted diet on ovarian apoptosis. (A) The levels of apoptosis in ovaries of females at 10 days postadult eclosion is compared among females fed pumpkin seed (dark gray bars) and sunflower seed (light gray bar). Error bars represent ± standard error. An example of a stained ovariole from a pumpkin-fed female (B) shows that positive apoptotic cells are evident in the germinarium of the ovariole.

To explore the potential physiological mechanism underlying response to the poor food environment, we compared levels of ovarian apoptosis in females fed the novel diet of pumpkin seed and those fed the adapted diet of sunflower seed. In*O. fasciatus*, each of the paired ovaries contains seven ovarioles. The anterior end of the ovariole contains the germarium with trophocytes, oogonia, and prefollicular tissues (Bonhag and Wick 1953). Posterior to the germarium is the vitellarium that typically contains three follicles that range in maturity from young (anterior) to older (posterior) follicles. Pumpkin-fed females had higher levels of ovarian apoptosis at 10 days (Fig. 4A;*F*_1,22_ = 36.664,*P*< 0.001). The green fluorescent dye that is indicative of apoptotic cells was observed mainly in the anterior germinarium and nutritive cords, rather than in the three developing oocytes (Fig. 4B).

### Effect of novel versus adapted diet on fecundity and life span

**Figure 5:**
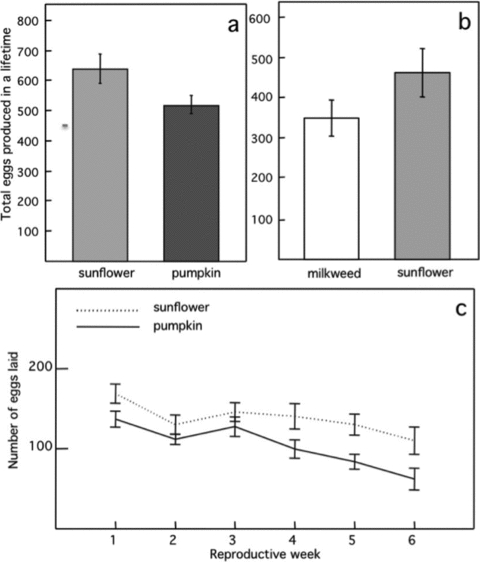
The effect of diets on egg production. The effect of pumpkin seed (dark bars) or sunflower seed (light bars) is compared for mean number of eggs produced over female's life span (A). The effect of milkweed seed (open bars) or sunflower seed (light grey bars) is compared for mean number of eggs produced over female's life span (B). The pattern of egg production between females fed pumpkin seed (solid line) and sunflower seed (dashed line) is compared among females that lived for at least 6 weeks postadult eclosion (C). Error bars represent ± standard error.

When all females were analyzed for total numbers of eggs laid over their life span, pumpkin-fed females laid fewer eggs than sunflower-fed females (Fig. 5A;*F*_1,75_ = 4.501,*P* = 0.037). The pattern of reproduction over time was examined on the subset of females that laid eggs over at least 6 weeks. In both pumpkin-fed and sunflower-fed females, the number of eggs produced changed over time (Fig. 5C; within subjects,*F*_5,17_ = 15.370,*P*< 0.001), although the change did not depend on the food type (within subjects, time × food*F*_5,17_ = 1.198,*P* = 0.352). There was a significant difference in the pattern of egg-laying between pumpkin-fed and sunflower-fed females (Fig. 5C; between subjects*F*_1,21_ = 10.539,*P* = 0.004). Specific pairwise comparisons showed no difference in reproductive output occurred in the first three weeks off egg production, but pumpkin-fed females had fewer offspring in weeks 4, 5, and 6 (Table 1). The overall result was that in this subset of females, for which the time available for reproduction is controlled, pumpkin-fed females laid fewer eggs over their life span than sunflower-fed females (Table 1).

**Table 1:** Specific pairwise contrasts between numbers of eggs laid by females across subsequent weeks of life. The data only include those females that lived for at least 6 weeks postadult eclosion

Egg production	*F*	*P*	Mean number of eggs laid (± SE)	
Week 1	*F*_1,21_ = 3.578	0.073	pumpkin	142.9 ± 9.3
			sunflower	168.9 ± 10.1
Week 2	*F*_1,21_ = 2.280	0.146	pumpkin	111.8 ± 8.0
			sunflower	130.1 ± 9.1
Week 3	*F*_1,21_ = 1.250	0.276	pumpkin	127.5 ± 10.9
			sunflower	145.9 ± 12.4
Week 4	*F*_1,21_ = 5.094	0.035	pumpkin	99.6 ± 11.9
			sunflower	140.4 ± 13.6
Week 5	*F*_1,21_ = 9.729	0.005	pumpkin	83.7 ± 9.8
			sunflower	130.1 ± 11.2
Week 6	*F*_1,21_ = 5.465	0.029	pumpkin	62.0 ± 13.6
			sunflower	110.1 ± 15.5
Total eggs laid	*F*_1,21_ = 10.539	0.004	pumpkin	621.5 ± 41.4
			sunflower	825.5 ± 47.3

**Figure 6:**
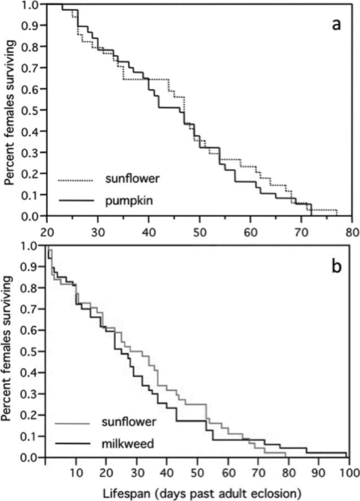
The effect of diets on female longevity. The survival curves for females fed pumpkin seed (dark gray line) and sunflower seeds (light gray line) are compared (A). The survival curves for females fed milkweed seed (dark gray line) and sunflower seeds (light gray line) are compared (B).

When all individuals were considered, there was no significant difference between survival of females fed the novel diet of pumpkin seeds and those fed the adapted diet of sunflower seeds (Fig. 6A; Wilcoxon χ^2^ = 0.022, df = 1,*P* = 0.881). While sample sizes are small for an accurate survival analysis, this was also the case for the females used in the repeated measures analyses (Wilcoxon χ^2^ = 0.608, df = 1,*P* = 0.436).

### Effect of ancestral versus adapted diet on fecundity and life span

The total number of eggs laid by milkweed- and sunflower-fed females over their lifetime was the same (Fig. 5B;*F*_1,49_ = 2.289,*P* = 0.137). There was no difference between survival of females fed milkweed seeds and those fed sunflower seeds (Fig. 6B; Wilcoxon χ^2^ = 0.562, df = 1,*P* = 0.454).

## Discussion

As predicted,*O. fasciatus*responded to a novel diet in a manner consistent with the oosorption hypothesis; resorbing oocytes and reallocating resources to somatic maintenance as evidenced by no reduction in life span, despite the poor quality of the novel diet. While overall our results support the hypothesis that oosorption via ovarian apoptosis is a mechanism by which females can respond to novel or poor food environments, the details of the impact of a novel food source on life-history traits indicated that the ability to respond is likely to be complicated by constraints of development and physiology. Our results provide evidence for a trade-off between current and future reproduction. This plastic response to food stress is common; poor nutritional environments trigger a physiological state geared toward survival at the expense of reproduction (Rion and Kawecki 2007). One mechanism proposed to underlie this trade-off is oosorption. Oosorption is considered to be an adaptive mechanism to conserve resources invested in eggs when conditions for reproduction are poor (Bell and Bohm 1975). It is presumed that these resources can then be allocated to survival until conditions for reproduction improve. While the positive correlation between oocyte resorption and survival has been documented in a few species, it is becoming clear that the trade-off between reproduction and survival is more complex than simple competition for limited resources (Partridge et al. 2005; Boggs 2009; Flatt 2011; Stearns 2011).

The population of milkweed bugs we have used in our study has had many generations under experimental evolution to adapt to a novel diet. Our results on life-history traits of this population on milkweed seed, the ancestral diet, compared to sunflower seed, the adapted diet, support our observation that this adaptation is an expansion of host range rather than a substitution, as has been seen in other species (e.g. Messina and Jones 2009). Females fed ancestral and adapted diets do not differ in fecundity or life span under laboratory conditions. It may be that the difference in time to sexual maturation, which is shorter on milkweed seeds, may have some advantage under natural conditions and highlights the need to be cautious about interpreting fitness effects in experimental populations in the laboratory.

The difference between adapted and novel diet affected a number of life-history traits. Females fed pumpkin seeds delayed producing offspring by one day. Females fed pumpkin seed also mate more often than those fed sunflower seed. Given that these females physiologically seem to have shifted away from a reproductive mode and toward a survival mode, it seems counterintuitive that they would invest in a reproductive behavior, which presumable reduced the amount of time available for foraging. Because of the way we did these experiments, it is impossible to separate out the effect of diet on the behavior of males and females. Thus, this increase in mating rate may be due to a diet effect on males. It is known in other lygaied bugs that mating influences female life history. In*Lygaeus equestris*, mating is costly and can affect both longevity and fecundity (Shuker et al. 2006). It is possible that in the lygeids these effects could be mediated via accessory gland proteins (Himuro and Fujisaki 2008), which could vary under different male diets.

While we did not observe any difference in egg quality based on development rates of embryos, we did observe a decrease in the number of eggs produced. This reduction in egg production was mirrored by an increase in ovarian apoptosis. However, our observations do not support a straightforward reduction in eggs laid through resorption of eggs that have initiated vitellogenesis. Apoptosis in the ovarioles was observed mainly in the region of the ovary where new oocytes are born rather than in oocytes undergoing vitellogenesis. Thus, the females do not appear to be “raiding the oocyte larder” for resources, but rather constraining future reproductive potential. This is also reflected in the pattern of reproduction observed between the pumpkin- and sunflower-fed females in which early fecundity is unaffected, but future clutches are smaller. This schedule of reproduction does not fit the “wait to reproduce” assumption of the oosorption hypothesis.

In many ways, the treatment of the novel food approximates dietary restriction, known to result in a shift in investment from reproduction to longevity (Partridge et al. 2005).*Oncopeltus fasciatus*presented with pumpkin seeds may simply choose not to feed, being highly specific in their food choice (Feir 1974). Alternatively they may feed but be unable to digest the constituent components. The transcriptome of the salivary gland from sunflower adapted*O. fasciatus*has been sequenced (Francischetti et al. 2007), and it would be interesting to compare the transcriptome of the salivary glands from adapted and ancestral populations to see if this is a target of selection.

It has recently been found that the trade-off between reproduction and longevity under dietary restriction can be broken in*Drosophila melanogaster*by supplementing with a single amino acid (Grandison et al. 2009). Mating status has also been recently shown to influence the longevity response to dietary restriction (Stoltz et al. 2010). Dietary restriction increases longevity in mated females but not unmated females, perhaps due to investment in mate-seeking behaviors being prioritized in unmated females. These studies and others showing variation in the response to poor nutritional environments (e.g. Carey et al. 2008) demonstrate that in order to understand how physiological and molecular mechanisms interact with internal and external factors in shaping the trade-off between reproduction and survival we need to examine these trade-offs in a variety of species (Stoltz et al. 2010; Flatt et al. 2011) in order to begin to develop the framework for predicting how shifts in diet availability will impact insect populations.
